# An electrochemically stable homogeneous glassy electrolyte formed at room temperature for all-solid-state sodium batteries

**DOI:** 10.1038/s41467-022-30517-y

**Published:** 2022-05-23

**Authors:** Xiaowei Chi, Ye Zhang, Fang Hao, Steven Kmiec, Hui Dong, Rong Xu, Kejie Zhao, Qing Ai, Tanguy Terlier, Liang Wang, Lihong Zhao, Liqun Guo, Jun Lou, Huolin L. Xin, Steve W. Martin, Yan Yao

**Affiliations:** 1 Department of Electrical and Computer Engineering and Texas Center for Superconductivity at the University of Houston, University of Houston, Houston, TX 77204 USA; 2 Shanghai Institute of Ceramics, Chinese Academy of Sciences, Shanghai, 200050 China; 3 Department of Materials Science & Engineering, Iowa State University, Ames, IA 50011 USA; 4 School of Mechanical Engineering, Purdue University, West Lafayette, IN 47907 USA; 5 Department of Materials Science and NanoEngineering, Rice University, Houston, TX 77005 USA; 6 Shared Equipment Authority, SIMS laboratory, Rice University, Houston, TX 77005 USA; 7 Department of Physics, Northern Illinois University, Dekalb, IL 60115 USA; 8 Department of Physics and Astronomy, University of California, Irvine, Irvine, CA 92697 USA; 9 Present Address: Institute of Advanced Structure Technology and School of Materials Science and Engineering, Beijing Institute of Technology, Beijing, 100081 China

**Keywords:** Batteries, Batteries

## Abstract

All-solid-state sodium batteries (ASSSBs) are promising candidates for grid-scale energy storage. However, there are no commercialized ASSSBs yet, in part due to the lack of a low-cost, simple-to-fabricate solid electrolyte (SE) with electrochemical stability towards Na metal. In this work, we report a family of oxysulfide glass SEs (Na_3_PS_4−*x*_O_*x*_, where 0 < *x* ≤ 0.60) that not only exhibit the highest critical current density among all Na-ion conducting sulfide-based SEs, but also enable high-performance ambient-temperature sodium-sulfur batteries. By forming bridging oxygen units, the Na_3_PS_4−*x*_O_*x*_ SEs undergo pressure-induced sintering at room temperature, resulting in a fully homogeneous glass structure with robust mechanical properties. Furthermore, the self-passivating solid electrolyte interphase at the Na|SE interface is critical for interface stabilization and reversible Na plating and stripping. The new structural and compositional design strategies presented here provide a new paradigm in the development of safe, low-cost, energy-dense, and long-lifetime ASSSBs.

## Abstract (web summary)

Single sodium-ion solid electrolyte that meets the requirements of practical applications is difficult to design. Here, the authors show how kinetic stability via the creation of a self-passivating solid electrolyte interphase allows a homogenous glass solid electrolyte to exhibit remarkable electrochemical stability with sodium metal.

## Introduction

Low-cost batteries with high safety and specific energy are in ever-increasing demand for grid-scale energy storage^1^. All-solid-state sodium batteries (ASSSBs) using nonflammable solid-state electrolytes (SEs) and earth-abundant sodium metal anodes are among the most promising candidates and therefore are attracting worldwide research attention^2–5^. So far, the only successful example of a commercialized Na metal anode battery for grid-scale energy storage is the well-known high-temperature sodium-sulfur battery^6^. At the high working temperature of >300 °C, both the Na anode and the S cathode are liquids, dramatically increasing the operational cost and decreasing safety due to potential fires and explosions caused by the catastrophic failure of the thin ceramic SE^7^. In contrast, ambient-temperature ASSSBs using solid Na metal anodes are significantly more desirable, not only because of their lower cost, but also because of their lower temperature operation, *T* < 100 ^o^C, which enables them to be more safely used in a broader range of applications. However, when operated such that the Na metal anode is now in solid state, not only must the SE be resistant to direct chemical and electrochemical reaction with Na, it must also be resistant to solid metallic sodium dendrite penetration. Therefore, the search for new SEs for ASSSBs must simultaneously meet the stringent requirements of low cost and facile fabrication, but also meet severe mechanical and chemical stability requirements. So far, no single sodium SE has been able to simultaneously meet all four of these requirements, and therefore, the development of SEs that are stable while cycling solid Na metal remains a great challenge.

**Fig. 1:**
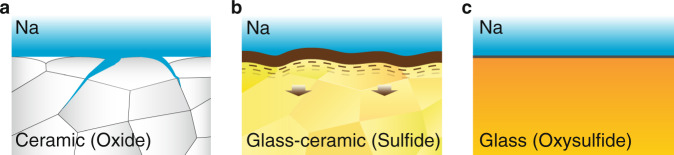
Classification of solid electrolyte−Na metal interfaces. **a** Na dendrites propagate along grain boundaries of oxide electrolytes; **b** decomposition of sulfide-based glass-ceramic electrolyte contacting Na metal; **c** homogeneous oxysulfide glassy electrolytes form stable interface with Na metal.

Inorganic SEs can be divided into three categories: ceramic, glass-ceramic, and glass. Ceramic SEs such as β″-Al_2_O_3_ and NASICON-type oxides exhibit excellent chemical stability towards Na metal. Nevertheless, their high Na-ion conductivities are achieved only when they are processed to near theoretical densities, requiring sintering temperatures in excess of 1500 ^o^C for long hours, and are subjected to poor wettability with Na metal due to their rigid and rough surface^8,9^. Furthermore, it has been observed that Na metal preferentially propagates along distinct grain boundaries, forming dendrites that eventually short circuit the electrolyte^10,11^ (Fig. 1a). This has been a source of controversy in the field of SEs because these ceramic oxide SEs have mechanical moduli in excess of 200 GPa and provide more than adequate elastic and shear moduli to resist Na dendrite. Glass-ceramic SEs (e.g., heat-treated Na_3_PS_4_, simplified as HT–Na_3_PS_4_) and other sulfide SEs have compliantly soft surfaces that exhibit less well-defined grain boundaries due to the existence of a certain quantity of a glassy phase (5–50 vol%), which can mitigate the dendrite formation and growth. It turns out, however, that when these SEs come into contact with Na metal, they break down into an unstable solid electrolyte interphase (SEI) layer^12–15^ (Fig. 1b). Because of these reasons, Na alloys like Na–Sn are often used as an anode. These alloys raise the voltage on the anode and decrease the energy density.

To date, there is no single SE that can simultaneously meet all of the mechanical, chemical, electrochemical, and processing requirements that can enable the ASSSBs with enhanced electrochemical performance and specific energy. We have recently reported the improved chemical stability of the mixed oxysulfide Na_3_PS_3_O SE in contact with metallic Na compared with pure sulfide SE^16^. Here, knowing the specific benefits of the individual SEs described above, and recognizing the inherent cost, process, and scale-up advantages of mechano-chemical milling (MCM), we report in this work a series of Na_3_PS_4−*x*_O_*x*_ oxysulfide glass SEs that consist of a predominant sulfide-based SE that is doped with a small amount, up to 15 mol.% of oxygen (*x* = 0.60), that can be rendered fully chemically reacted from simple precursors by MCM and fully glassy and homogeneous by pressure sintering at room temperature.

These new glass SEs begin as fine-grained and agglomerated powders as a result of the MCM processing and feature properties and structures that are strongly dependent on the oxygen doping. We have observed that during normal uniaxial pressing at pressures less than 300 MPa, these SEs spontaneously transform into fully dense and microscopically and macroscopically homogeneous smooth glasses that behave as if they had been melt quenched (MQ) to the normal glassy state. Their homogeneous structures appear to be isostructural to glasses of the same composition produced by normal MQ processes. This structural transformation appears to be unique among oxysulfide SEs at the reported oxygen doping levels. We have found that the formation of the homogenous bulk glassy phase is an essential property of these SEs in order to be mechanically, chemically, and electrochemically stable against Na metals (Fig. 1c).

The ternary composition of Na_2_S–P_2_S_5_–P_2_O_5_ reported here has been carefully designed according to five rules: first, P_2_S_5_ is a strong glass former that appears to facilitate the formation of a structurally homogeneous bulk glass, which we find is crucial to suppressing the Na dendrites propagation^17,18^. Second, following previous studies of oxysulfide glasses where the added oxygen preferentially forms structure enhancing bridging oxygen (BO), the glass network can be reinforced with stronger chemical bonding of P–O compared to that of P–S^19^, thereby boosting the mechanical strength^15,20,21^. Third, following our previous observations on binary Na_2_S + P_2_S_5_ glasses^22,23^, made either by MCM or MQ techniques, maximum Na-ion conductivity was observed at maximum Na_2_S concentration. Fourth, pure sulfide glasses always exhibit superior Na-ion conductivities over pure oxide glasses^24–27^. And fifth, again following our previous observations^28^ and those of Hayashi et al^29^., that in mixed oxysulfide glasses, the conductivity rapidly increases with small sulfur replacements, <25 mol.%, but then decreases after this maximum.

In this work, we conducted a systematic characterization of the sodium phosphorous oxysulfide SE, Na_3_PS_4−*x*_O_*x*_ (NPSO), where 0 ≤ *x* ≤ 0.60, by examining their chemical structures, mechanical properties, the composition and thickness of SEI formed at the SE–Na interface, and their overall electrochemical performance as measured in Na|SE|Na symmetric cells. Their performance at both reducing and oxidizing potentials was examined by forming and successfully cycling ambient-temperature sodium-sulfur batteries. We believe these mixed oxysulfide SEs and their unique homogenous bulk glassy structure produced by room-temperature pressing provide a new paradigm in the development of safe, low-cost, energy-dense, and long-lifetime ASSSBs.

## Results and discussion

### Formation of chemically reacted glasses

The synergistic effects of oxygen additions on the properties of the NPSO SEs were examined for different compositions of *x* = 0, 0.15, 0.30, and 0.60 that were synthesized via the high-energy MCM and systematically characterized. The XRD patterns (Supplementary Fig. S1) show that all the raw materials became amorphous after MCM, as no diffraction peaks of the starting materials were detected. In Hayashi et al.’s and our previous studies, unreacted Na_2_S and P_2_S_5_ were observed for MCM time shorter than those used here and unreacted Na_2_S was observed for Na_2_S contents higher than those used here^22,30^. Chemical spectroscopies, infrared (IR), Raman, nuclear magnetic resonance (NMR), and X-ray photoelectron spectroscopy (XPS) were used to demonstrate the full chemical reaction between Na_2_S and P_2_S_5_, rather than just amorphization of the starting materials. The DSC curves in Supplementary Fig. S2 further reveal that all of the amorphous samples are glassy, exhibiting a glass transition (*T*_*g*_) around 200 °C. It needs to be pointed out that, for all of the glass samples, the glass formers are relatively poor and readily crystallize upon heating only slightly above the *T*_*g*_ of the glass. This rapid crystallization “cuts off” the full observation of the glass transition in these glasses and, as such, causes the *T*_*g*_ to be a very weak signature. The DSC trace of the *x* = 0.15 glass shows this effect of crystallization right after entering the glass transition. However, for the oxysulfide SEs (*x* ≥ 0.30), the working range, or the difference between *T*_*g*_ and *T*_*c*_, becomes larger with increasing oxygen content, indicating adding oxygen results in greater resistance to crystallization.

While a complete understanding of the complex mechano-chemical processes taking place during high-energy MCM is yet not fully known, it is generally agreed that MCM generates high local temperatures during the rapid ball-to-material-to-wall and ball-to-material-to-ball collisions and generates rapid thermal quenching after the collision as the materials are thermalized back to the near room temperature of the reaction vessel. The combination of high local temperature and rapid quenching is believed to be responsible for the formation of amorphous materials that also exhibit a liquid state glassy *T*_*g*_. However, just as in MQ systems, the quenching rate is not always sufficient to yield a fully amorphous glass and the presence of small amounts of crystalline phases can be detected in some systems, which was recently observed in the Na_2_S–P_2_S_5_ system^31^ and the Li_2_S–P_2_S_5_ system^32^.

**Fig. 2:**
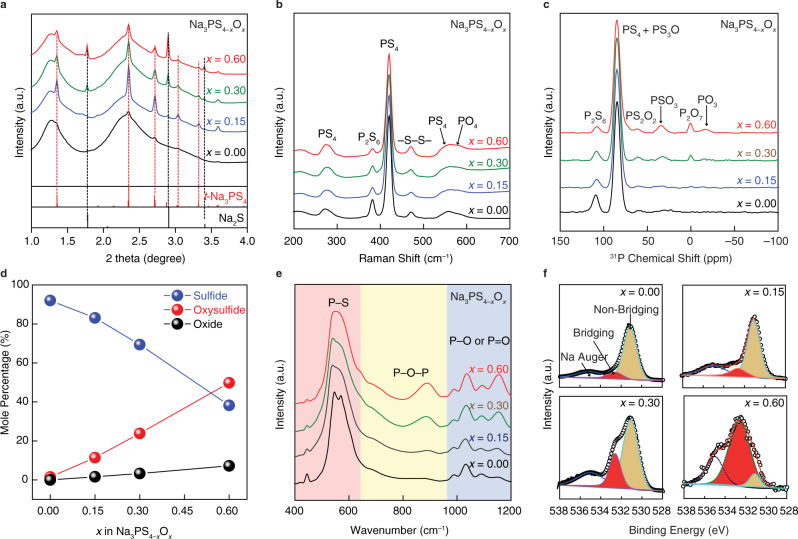
Structural characterizations of amorphous Na_3_PS_4−*x*_O_*x*_ (*x* = 0, 0.15, 0.30, and 0.60) SEs. Spectra from **a** Synchrotron X-ray diffraction, **b** Raman, **c** ^31^P NMR, **d** Structural units and their relative mole percentage, **e** Fourier transform-infrared spectra (FTIR), and **f** O 1 *s* X-ray photoelectron (XPS).

To examine in more detail the possible formation of small amounts of fine-grained crystalline phases in the NPSO SEs during the MCM process, high-energy synchrotron XRD patterns were collected and shown in Fig. 2a. All synthesized SEs feature two broad halos superimposed with weak Bragg peaks, indicating that a small number of fine-grained crystalline phases do form and are embedded in the majority of an otherwise glassy matrix. The SEM images (Supplementary Fig. S3) of the *x* = 0.15 SE show that the major glassy powders are distributed in the range of 2 to 8 μm with an average particle diameter of 5.3 μm. The TEM image and FFT pattern (Supplementary Fig. S4) further reveal that the crystalline phase in the glassy matrix is tetragonal Na_3_PS_4_ (*t*−Na_3_PS_4_). However, as seen in Fig. 2a, when *x* reaches 0.60, these crystallization processes weaken, as evidenced by the fact that the *t*−Na_3_PS_4_ peak has almost disappeared. However, it is now replaced by the appearance of crystalline Na_2_S that may arise from an incomplete chemical reaction.

The reason for the appearance of Na_2_S at the higher O substitution was examined by Raman spectroscopy since it is sensitive to sulfur-containing units. According to Fig. 2b, the main feature peak^25^ at 420 cm^−1^ is the stretching mode of the PS_4_ unit, which is a non-bridging sulfur (NBS) unit, identified as P^0^ unit^24,30^ where the superscript 0 is the number of bridging oxygens (BOs) on this short range order (SRO) unit. The Gaussian fitting of this mode (Supplementary Fig. S5) suggests that with the incorporation of oxygen, a small population of the original P^0^ units disproportionate into P^1^ units (containing one bridging sulfur (BS), e.g., P_2_S_7_) with the liberation of sodium sulfide (2P^0^ → 2P^1^ + Na_2_S)^24^ to balance charge. This disproportionation reaction is supported by the observation of Na_2_S peaks in the synchrotron XRD results. The mode centered at 380 cm^−1^ is assigned to the P_2_S_6_ unit, which possesses a homopolar P–P bond that decreases in concentration with the oxygen addition^33^. The formation process of the P_2_S_6_ defect unit generates residual sulfur species (e.g., sulfur element) that show a characteristic Raman mode arising from the –S–S– linkage at 470 cm^−1^. As indicated from both Fig. 2a, b, except for a small amount of crystalline phases of Na_3_PS_4_ and Na_2_S, which we estimate to be in the range of <5 mol.%, the main composition of the NPSO SEs is glassy.

### Chemical short-range order

^31^P MAS–NMR was used to gain further insights into the glassy phase of these NPSO SEs by examining the local structure around the phosphorus glass-forming cation. The deconvolution of the ^31^P NMR spectra (Fig. 2c) shows that the glass Na_3_PS_4_ (*x* = 0) is composed mainly of PS_4_ (82 ppm) and P_2_S_6_ (108 ppm) units, which is consistent with the Raman spectra described above. With the incorporation of oxygen, three new peaks attributed to the formation of mixed oxysulfide and oxide units, PS_2_O_2_ oxysulfide (63 ppm), PSO_3_ oxysulfide (32 ppm), and P_2_O_7_ oxide (3 ppm), can be clearly observed^34^. The peak for the PS_3_O oxysulfide units that was supposed to be there but is almost impossible to see because it has almost the same chemical shift as the PS_4_ units^29^.

However, following the methods we proposed before^24,35^, this unit and its relative fraction of all of the SRO units were determined by careful spectral deconvolution and corrected for charge balance among the Na^+^ ions and the various PS_4−*x*_O_*x*_ anions. The composition dependence of the population of the various SRO structural units in these oxysulfide NPSO SEs is given in Fig. 2d and shows that, as expected, the fractions of mixed oxysulfide units dramatically increase with oxygen doping level and become the dominant species when *x* reaches 0.60. The appearance of PS_4−*x*_O_*x*_ oxysulfide SRO units suggests that oxygen has been incorporated into the P–S tetrahedral unit. Due to the much stronger chemical bonding of P–O compared to that of P–S^19^ and the bridging oxygens that can microstructurally connect the units to a more robust and homogeneous glass network, the mechanical strength of the SEs after oxygen incorporation were expected to be improved.

FTIR spectroscopy was further applied to explore the chemical bonding between phosphorus and sulfur and phosphorous and oxygen. The FTIR spectra shown in Fig. 2e can be divided into three parts corresponding to terminal P–S^−^ (400–600 cm^−1^), BO P–O–P (600–950 cm^−1^) and terminal non-bridging oxygens (NBOs) P–O^−^ or P = O (950–1200 cm^−1^) modes, respectively^36,37^. Detailed peak assignments are listed in Supplementary Table S1. It is evident that oxygen incorporation into the glass matrix leads to a slightly increased fraction of P–O^−^ and P = O bonds and a particularly significant increase in the fraction BO P–O–P bonds, where the O atom is bridging between two phosphorus atoms. Further evidence of the formation of BOs on adding oxygen to Na_3_PS_4_ can be found in O 1 *s* XPS spectra (Fig. 2f), showing the BO with a binding energy of 532.5 eV^38,39^ accounts for the higher fraction of the oxygen for larger *x* values in NPSO SEs. Spectral deconvolution of Na_3_PS_3.4_O_0.6_, for example, shows that more than 90% of the added oxygen atoms are present as BOs in the glass. This behavior is completely consistent with our previous work^40^ where in a similar Li_2_GeS_4−*x*_O_*x*_ glass, the added oxygen was also found to preferentially replace S in the bridging sulfur (BS) positions until all of the BSs were eliminated. The oxygen did not form NBOs until there were no more BSs to replace. The results here for the NPSO series reveal a more complex mechanism for the added oxygen to form BO because in the present system, there are no BSs to begin with. Hence, the added O forces the formation of the disproportionation reactions described above. To maintain charge balance in the series, the formation of a BO requires the formation of “free” Na_2_S in direct proportion to the amount of oxygen added. While overall charge balance appears to be maintained in this system, the formation of BOs through the addition of oxygen also forms increasing chain units in the form of P^1^ units. We have observed this same behavior in the slightly lower modifier Na_4_P_2_S_7−*x*_O_*x*_ system^35^.

### Homogeneous glass with improved mechanical properties

**Fig. 3:**
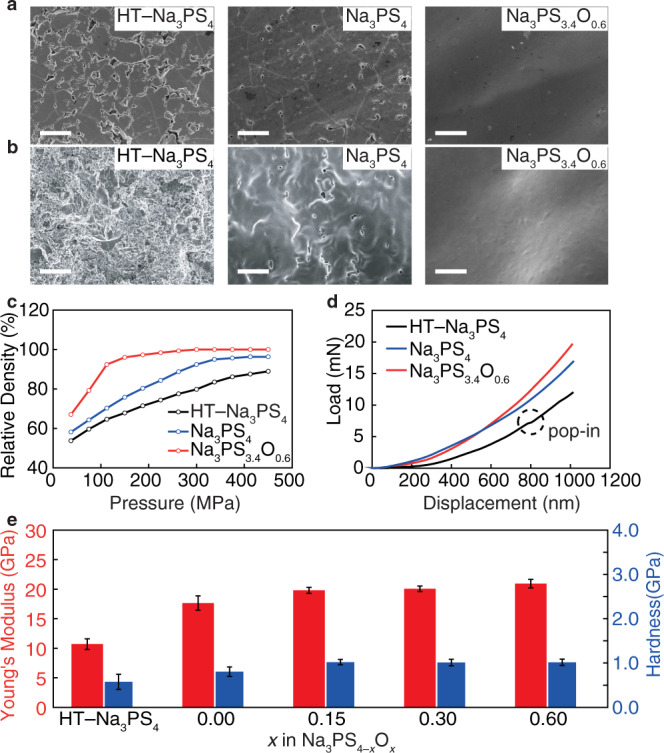
Mechanical properties of Na_3_PS_4−*x*_O_*x*_ glass SEs (*x* = 0 and 0.60) and HT–Na_3_PS_4_ glass-ceramic SE. **a** Surface and **b** cross-sectional morphology, scale bar 10 μm; **c** Relative density vs. fabrication pressure; **d** Nanoindentation loading curves; error bars indicate the standard deviation of nine tests; and **e** Young’s modulus and hardness of various SEs. HT–Na_3_PS_4_ represents the heat-treated Na_3_PS_4_ glass-ceramic electrolyte.

A homogeneous and mechanically robust SE is a prerequisite for successfully cycling with Na metal anodes^11^. Consequently, the morphological structure and the mechanical properties of NPSO SEs were investigated. For comparison, the widely studied heat-treated Na_3_PS_4_ glass-ceramic SE (HT–Na_3_PS_4_) was also studied^25,41^. Pores and grain boundaries are clearly observed in the SEM images of pelletized HT–Na_3_PS_4_ (Fig. 3a, b). These surface defects are believed to induce dendrite formation, subsequent dendrite propagation^42–44^, and eventual short-circuit through the SE, as demonstrated in Supplementary Fig. S6. Na_3_PS_4_ glass SE, however, shows fewer structural pores compared to HT–Na_3_PS_4_. In sharp contrast to both of these SEs, the NPSO glass SE appears to be free of pores from the surface through to the interior.

To the best of our knowledge, this fully homogeneous and dense glassy morphology induced by simple and low-cost room temperature uniaxial pressing is observed here for the first time in any SE fabricated by simple cold-pressing. Such a morphology is presumably closely related to the outstanding formability of NPSO. As shown in Fig. 3a, b, and Fig. S7, this phase is nearly fully densified at a pressure as low as 150 MPa. In comparison, Na_3_PS_4_ glass and HT–Na_3_PS_4_ couldn’t achieve a similar relative density even when much higher pressures were applied, as seen from Fig. 3c. The excellent formability of these oxysulfide SEs is attributed to the synergistic effects of mixed P_2_S_5_ and P_2_O_5_ glass formers, which not only build a close-packed glass network with abundant BO units but also facilitate the local sintering process of the powders as demonstrated by more interparticle adhesion and necking compared to the pure sulfide SEs particles (Supplementary Fig. S8). Overall, for oxysulfide SEs, their unique formability and homogeneous structure will undoubtedly enhance their mechanical strengths and reduce the likelihood of dendrite-induced short-circuit when using the Na metal anode, as described in Fig. 1a.

To quantify the mechanical properties of SEs, two critical parameters: Young’s modulus *E* and hardness *H* were measured using the nanoindentation technique^45^. Typical load–displacement curves in Fig. 3d and Supplementary Fig. S9 show that the HT–Na_3_PS_4_ pellet experiences a sudden increase in indenter penetration during the loading process, which is not found in glassy NPSO SEs. This is referred to as “pop-in” and is associated with the generation of a crack^45,46^. This behavior indicates that the HT–Na_3_PS_4_ SE is more brittle than its glassy counterparts. Such a cracking is certainly unfavorable for the preparation and practical application of SEs. Further, benefiting from the homogeneous property of the glassy materials, the NPSO SEs display very small standard deviations for *E* and *H* as seen in the bar chart in Fig. 3e. Moreover, oxygen doping results in an increase of *E* and *H* of SEs, which supports the observation that oxygen doping produces more BO units in the glass network, thus providing more homogeneous and robust mechanical properties. The *E* and *H* of Na_3_PS_3.4_O_0.6_ glass were measured to be 20.9 ± 0.7 GPa and 1.0 ± 0.1 GPa, respectively, which are the highest among the NPSO series and even higher than those of the reported sulfide-based Li-ion and Na-ion SEs prepared by hot-pressing^18,47,48^. Assuming that Poisson’s ratio *ν* is ca. 0.3 according to the study by Sakuda et al^49^., the shear modulus *G* of Na_3_PS_3.4_O_0.6_ glass is ca. 8.0 ± 0.3 GPa, which is believed to be sufficient to suppress dendritic penetration of Na metal as predicted by the Monroe and Newman criterion^50^.

### Characterization of the Na−SE interface

**Fig. 4:**
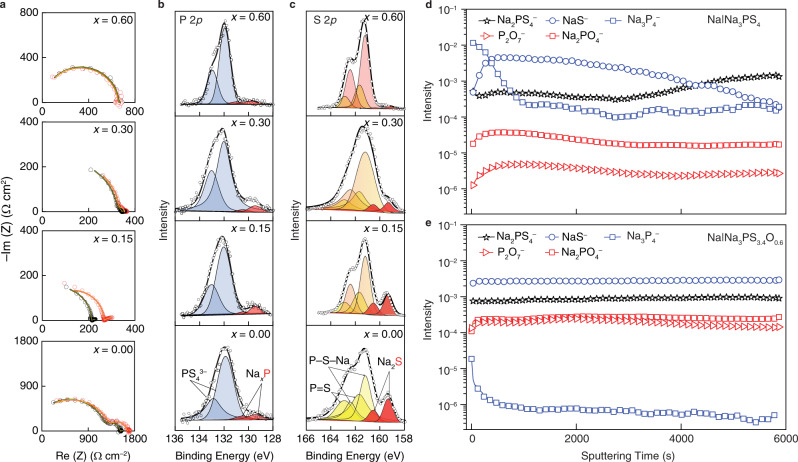
Chemical stability of Na_3_PS_4−*x*_O_*x*_ (*x* = 0, 0.15, 0.30, and 0.60) SEs towards Na metal at 60 °C. **a** Electrochemical impedance spectra (EIS) evolution of freshly made Na|SE |Na symmetric cells (black curve) vs after 5 h of rest (red curve); XPS spectra of **b** P 2*p* and **c** S 2*p* for the interface between Na and SE; **d**, **e** Depth profiles of fragments from the ToF-SIMS spectra of the Na|Na_3_PS_4_, and Na|Na_3_PS_3.4_O_0.6_ interfaces after 5 h of contact.

We first examined the Na−NPSO interface using time-dependent electrochemical impedance spectroscopy (EIS) and XPS. Na|NPSO|Na symmetric cells were fabricated to monitor the evolution of the EIS spectra and the area specific resistance (ASR) over a period of 5 h of resting and those are shown in Fig. 4a and Supplementary Fig. S10, respectively. The obtained EIS spectra can be divided into three semicircle regions^51^, and the fitting parameters are listed in Supplementary Table S2. The high-frequency semicircle represents the combined bulk and grain boundary resistance and capacitance (R_b_ + R_gb_, C_b_ + R_gb_) of the SEs; the mid-frequency semicircle with the characteristic capacitance of 10^−6^~10^−7^ F arises from the interfacial resistance and capacitance (R_i_, C_i_) of SEI formed between Na and SE^52^. As seen from Table S2, the added oxygen has a positive effect on improving the observed stability against Na metal. Fig. S10 shows that the change of ASR becomes less significant when more oxygen is doped.

To identify the composition of SEI that apparently grows with time for the *x* ≠ 0.60 glassy NPSO SEs, Na metal was detached from the symmetric cells and the surface of SEs was probed by the XPS. Compared to the surfaces of the pristine SEs (Supplementary Fig. S11), Fig. 4b, c shows that the surfaces of the Na_3_PS_4−*x*_O_*x*_, *x* = 0, 0.15, and 0.30, SEs exhibit two new pairs of doublets for P 2*p* spectra and S 2*p* spectra at lower bonding energy after contacting with Na metal. These new pairs of doublets correspond to the reduced phosphide and sulfide species, respectively. According to the theoretical calculation from Tian et al^12^. and the experimental study from Wenzel et al.^13^, these reduced species are Na_2_S and Na_3_P, respectively, the latter of which is a mixed ionic and electronic conductor that can cause continuous reduction of the SE. Similar effects have been found in other pure sulfides and selenides, such as Na_3_PS_4_, Na_3_SbS_4_, Na_3_PSe_4_, and while they all have high ionic conductivities, their tendency to produce these unstable SEIs renders them unsuitable for use in Na metal batteries^14^. In contrast, Fig. 4b, c shows that these same XPS signals from these reduced species are nearly indistinguishable above the baseline noise in the S 2*p* and P 2*p* spectra for the Na_3_PS_3.4_O_0.6_ SE.

We used the Time-of-Flight Secondary Ion Mass Spectrometry (ToF-SIMS) to better understand the composition of the SEI layer at the Na|SE interface. The selected mass spectra of the surfaces of the two solid electrolytes (Na_3_PS_4_ and Na_3_PS_3.4_O_0.6_) after Na metal removal are shown in Supplementary Fig. S12. Two strong peaks at 55.0 m/z and 192.8 m/z correspond to NaS^−^ and Na_3_P_4_^−^ fragments, respectively, in the Na|Na_3_PS_4_ sample (black). The depth profile of these two fragments during Cs^+^ ion sputtering is shown in Fig. 4d. It is reasonable to assign the two fragments to Na_2_S and Na_3_P as a result of Na_3_PS_4_ reduction: 8Na + Na_3_PS_4_ → 4Na_2_S + Na_3_P. We estimate the thickness of the SEI based on the profile of Na_3_P_4_^−^ fragment because Na_3_P is electronically conductive, which dictates the extent of electrolyte decomposition. The thickness of the SEI in the Na|Na_3_PS_4_ sample corresponds ~1000 s of Cs^+^ sputtering. In contrast, the intensity of Na_3_P_4_^−^ at surface (~10^−5^ in the Na|Na_3_PS_3.4_O_0.6_ sample) is three orders of magnitude lower than in the Na|Na_3_PS_4_ sample (~10^−2^). Furthermore, the intensity drops to 10^−6^ after only 100 s of sputtering. This comparison shows, in the case of the Na|Na_3_PS_3.4_O_0.6_ sample, the initial reduction of SE is suppressed by a factor of 1000 and the thickness of SEI layer is reduced at least by a factor of ten. After initial SEI formation, the interface stabilizes and Na metal plating process initiates. We hypothesize that the self-passivating nature of Na_3_PS_3.4_O_0.6_ decomposition products is owing to the formation of electrically insulating Na_2_O, the signal of which is too weak to detect in the ToF-SIMS spectra due to the negligible SE reduction. Other fragments in Fig. 4e, including P_2_O_7_^−^ (174.0 m/z), Na_2_PO_4_^−^ (140.9 m/z), and Na_2_PS_4_^−^ (204.8 m/z), are all derived from the bulk of Na_3_PS_3.4_O_0.6_, which exhibits flat profiles during Cs^+^ sputtering. To our knowledge, this is the first sulfide-based fully homogeneous glassy SE that produces a self-passivating, thin SEI against Na metal.

### Electrochemically stable tri-layer composite SEs

**Fig. 5:**
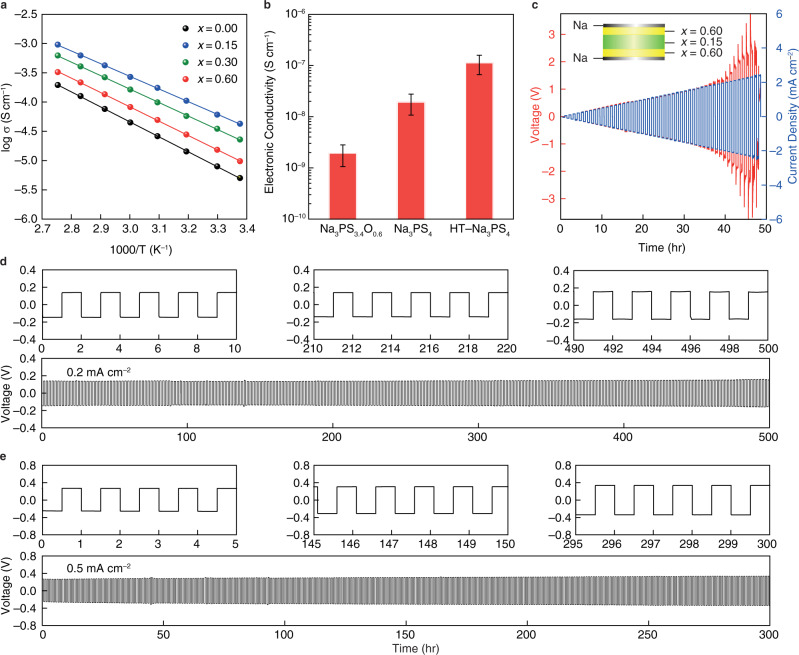
Electrochemical properties of glassy oxysulfide SEs. **a** Temperature dependence of the Na-ion conductivities of SEs; **b** Electronic conductivity of three types of SEs measured by two-blocking electrodes at 60 °C, error bars indicate the standard deviation of three tests; **c** Critical current density (CCD) test of a tri-layer SE in Na|SE|Na symmetric cell (SE is Na_3_PS_3.4_O_0.6_|Na_3_PS_3.85_O_0.15_|Na_3_PS_3.4_O_0.6_) at 60 °C. The step current density increase is 0.05 mA cm^−2^ and plating/stripping period is 0.5 h each; **d**, **e** Galvanostatic cycling of the symmetric cells at 60 °C with current densities of 0.2 mA cm^−2^ and 0.5 mA cm^−2^, respectively. HT–Na_3_PS_4_ represents the heat-treated Na_3_PS_4_ glass-ceramic electrolyte.

Figure 5a shows the temperature-dependence of the Na^+^ ionic conductivities that were obtained from the Nyquist plots (Supplementary Fig. S13) for the Na_3_PS_4−*x*_O_*x*_, *x* = 0, 0.15, 0.30, 0.60. From Supplementary Fig. S14, it is intriguing that the ionic conductivities exhibit a 6-fold increase with the initial addition of oxygen, *x* = 0.15, rendering the conductivity of the Na_3_PS_3.85_O_0.15_ SE as high as 2.7 × 10^−4^ S cm^−1^ and activation energy as low as 41.5 kJ mol^−1^. This unusual rise in ionic conductivity could be attributed to two unique effects of the addition of oxygen to the base Na_3_PS_4_ glassy SE. First, in a recent study^28^, we found that doping oxygen into the comparable Li_2_GeS_2−*x*_O_*x*_ glassy SE enhanced the free volume available to the mobile Li^+^ ion for conduction. As a result, the ‘doorway’ radius, r_D_, between Li^+^ ion sites increased, lowering the overall activation energy and increasing the conductivity^28^. The compositional similarity and the similar sharp increase in the conductivity suggest a similar underlying mechanism for Na_3_PS_4−*x*_O_*x*_ and Li_2_GeS_2−*x*_O_*x*_ glassy SEs. Second, as shown by the synchrotron XRD data in Fig. 2a, the presence of a highly conductive *t*−Na_3_PS_4_ crystalline phase^25^ may introduce a second highly conductive parallel phase in the glass matrix, albeit in a less than 5 mol.% fraction, resulting in enhanced overall conductivity^53^.

Additions of oxygen monotonically increase the activation energy thereby decreasing the conductivity. Consistent with our previous studies^28^, the added oxygen shrinks the structure, reducing the free volume available for conduction and boosting activation energy through the formation of more BO units. Further decreasing the conductivity is the formation of a very poorly conductive Na_2_S phase formed through the disproportionation reaction described above. As a result, at 60 °C, the Na_3_PS_3.4_O_0.6_ SE has a reduced conductivity of 8.2 × 10^−5^ S cm^−1^. Furthermore, it has been recently reported that high electronic conductivity of SEs, as well as internal defects (cracks, pores, grain boundaries) are responsible for the creation and propagation of Li dendrites in SEs^54,55^. Figure 5b compares the electrical conductivity of Na_3_PS_3.4_O_0.6_, Na_3_PS_4_, and HT–Na_3_PS_4_ using ion-blocking electrodes. The electrical conductivity of Na_3_PS_3.4_O_0.6_ is two orders of magnitude lower than that of HT–Na_3_PS_4_, suggesting the unique homogeneous microstructure of oxysulfide SE, which will surely reduce the chance of dendrite-induced short-circuit.

However, the excellent formability of the Na_3_PS_3.4_O_0.6_ SE allows for the fabrication of a thin SE layer with very low resistance. A perfect SE for ASSSBs requires both high ionic conductivity as well as excellent mechanical and chemical stability. To produce such an SE, a tri-layer architecture was designed with the most ionically conductive Na_3_PS_3.85_O_0.15_ in the inside and the mechanically and chemically stable Na_3_PS_3.4_O_0.6_ on the outside, as shown in the inset of Fig. 5c. Supplementary Fig. S15 shows a fully all-glass SE separator without any discernible voids or grain boundaries was attained owing to the excellent formability and densification. The cyclability of the tri-layer electrolyte was investigated in a symmetric Na|SE|Na cell using gradient-current and constant-current experiments, as illustrated in Fig. 5c–e. The voltage profile of the tri-layer separator shows an ohmic response, V = IR; however, sudden voltage spikes occur when the current exceeds 2.3 mA cm^−2^, which was estimated to be the critical current density (CCD) for the tri-layer SE. The highest recorded CCD value for sulfide-based Na-ion SEs is 2.3 mA cm^−2^, which is also equivalent to the state-of-the-art CCD value of lithium-based sulfide SEs. The improved capability to resist Na dendrite formation and penetration is ascribed to their homogeneous and porosity-free structure, as well as their robust mechanical properties. These findings are consistent with Porz et al.’s^11^ conclusions on the failure processes of SEs toward a metal anode, in which they discovered that Griffith’s flaws in the SEs cause dendritic growth and propagation across SEs.

At current densities of 0.2 mA cm^−2^ and 0.5 mA cm^−2^, symmetric cells with tri-layer electrolytes can cycle for hundreds of hours without short-circuiting, as shown in Fig. 5d, e. The inserts to Fig. 5d, e show that remarkably flat voltage profiles are recorded at each cycle over the duration of the cell’s cycling. This behavior suggests that the Na plating and stripping processes are quite stable at the Na_3_PS_3.4_O_0.6_|Na interface, which is consistent with the previous explanation. The charge-transfer resistance for Na_3_PS_3.4_O_0.6_|Na is only 10 Ω cm^2^. Supplementary Fig. S16 compares this study to some state-of-the-art findings on Na|SE|Na symmetric cells for various kinds of known Na-ion conducting SEs. This figure depicts three key parameters that influence the energy density, power density, and cycle life of full cells: the capacity of Na metal-plated per cycle (area of the circle), current density, and cycling time. It is clear that the composite tri-layer oxysulfide SE produced in this work greatly improves the cycle duration and current density performance of SEs cycling Na in symmetric cells. It is worth noting that the mass transport in Na metal is sufficient to support stable cycling without the introduction of interfacial voids^56^. In particular, our composite tri-layer SE sets new standards for higher current density and longer cycling duration in ASSSBs.

As previously demonstrated, the Na_3_PS_3.4_O_0.6_ SE can be coupled with Na_3_PS_3.85_O_0.15_ to form a stable all-glass separator. Similarly, by employing a tri-layer design, Na_3_PS_3.4_O_0.6_ can be used for couple with glass-ceramic SEs, e.g., HT–Na_3_PS_4_, or Na_3_SbS_4_. Supplementary Fig. S17 shows an example of this for Na_3_PS_3.4_O_0.6_|HT–Na_3_PS_4_|Na_3_PS_3.4_O_0.6_. Excellent interfacial contact may be formed between these two SEs due to the ease with which the Na_3_PS_3.4_O_0.6_ SE deforms under pressure to form a homogeneous interface. Because HT–Na_3_PS_4_ SE has a comparable conductivity (3.9 × 10^−4^ S cm^−1^)^57^ to Na_3_PS_3.85_O_0.15_ SE, Fig. S17 demonstrates that the Na|tri-layer SE|Na symmetric cells have a very similar overpotential to the symmetric cells illustrated in Fig. 5d, e. Furthermore, stable Na plating/stripping profiles at 0.2 mA cm^−2^ were achieved for up to 500 h and up to 240 h at 0.5 mA cm^−2^.

### All-solid-state Na–S full cells

The excellent Na_3_PS_3.4_O_0.6_|Na stability enables the fabrication of ASSSBs, of which the ambient-temperature Na–S battery is one of the most promising because of its very low cost and high theoretical specific energy. On the basis of the above study that demonstrated the stability of the tri-layer SE in a Na metal symmetric cell, a Na–S battery having the architecture of S–Ketjen Black (KB)–Na_3_PS_3.85_O_0.15_|Na_3_PS_3.85_O_0.15_|Na_3_PS_3.4_O_0.6_|Na was designed and tested.

**Fig. 6:**
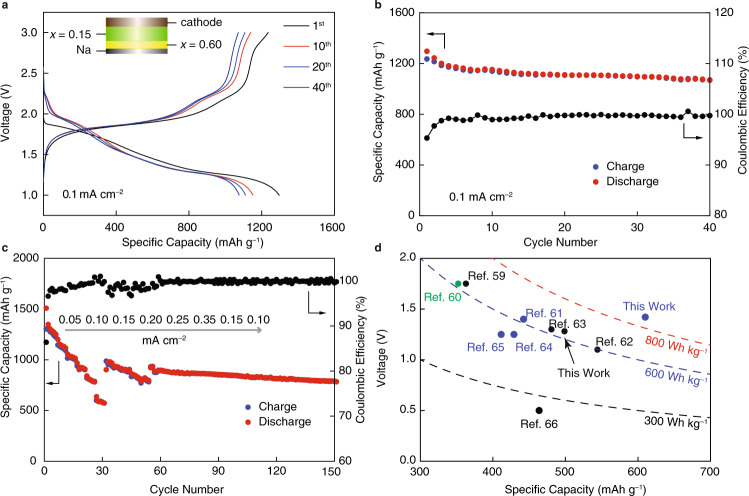
All-solid-state Na–S full cells measured at 60 °C. **a** Charge-discharge voltage profiles and **b** Capacity and coulombic efficiency vs. cycle number at a current density of 0.1 mA cm^−2^; **c** Rate capabilities of the Na–S full cell at various current densities (0.05–0.35 mA cm^−2^); **d** Summary of cell voltage–full cell capacity plot and material-level specific energy for reported ambient-temperature solid-state Na–S batteries (Room temperature: black; 60 ^o^C: blue; 90 ^o^C: green). Fig. S18 depicts the performance of a Na–S battery based on the oxysulfide SEs at ambient temperature. See Supplementary Table S3 for details. It is worth noting that the specific capacity in Fig. 6d depicts full-cell capacity while accounting for both the cathode and the anode.

The voltage profiles for the cell operating within the voltage window of 1.0 to 3.0 V are shown in Fig. 6a. The cell has a high initial discharge capacity of 1280 mAh g^−1^, which is 76% of the theoretical capacity of sulfur (Na→Na_2_S: 1675 mAh g^−1^) and substantially higher than that the high-temperature Na–S battery^58^, which has a capacity of 558 mAh g^−1^. This is due to the fact that the Na–S battery presented here is an all-solid-state system without the discharge limit of Na→Na_2_S_4_ that the high-temperature Na–S battery has. The first cycle coulombic efficiency is 92%, indicating that the polysulfide shuttle phenomenon, which is frequent in liquid electrolyte-based cells, is not observed. After the first five cycles, the cell achieves ~100% coulombic efficiency. After 40 cycles, the reversible capacity stabilizes at around 1000 mAh g^−1^ with a capacity retention of more than 80% (Fig. 6b). These values are much higher than those reported for Na–S batteries using oxide or polymer SEs and Supplementary Table S3 compares these previously reported solid-state Na–S batteries^59–66^ operating near ambient temperature to those described here. The discharge voltage profile consists of two plateaus at 1.9 V and 1.25 V vs. Na/Na^+^, with a single slope in between. The average discharging potential is 1.42 V, which is higher than the discharging potentials of existing pure sulfide SE-based Na–S batteries with Na_15_Sn_4_ or Na_3_Sb alloy anodes.

The rate capability of our Na–S full cells was examined by cycling at increasing current densities. The cell can offer specific capacities of 1116, 908, and 574 mAh g^−1^ at current densities of 0.10, 0.20, and 0.35 mA cm^−2^, respectively as seen in the Supplementary Fig. S19. After the current was reduced to 0.10 mA cm^−2^, the cell capacity restored to near-original levels, and the cell cycled stably for 150 cycles (Fig. 6c). The significantly improved Na–S battery cycling performance is attributed to the excellent interface stability, which allows Na metal to plate/strip at high rates, the superior formability property of oxysulfide SEs, which ensures the consistently good contact with sulfur and carbon during cycling, and the overall solid nature of the SEs, which prevents any polysulfide shuttle between the anode and the cathode. As a result, among all currently reported all-solid-state Na–S batteries, the oxysulfide-based Na–S battery described here has the highest specific energy (Fig. 6d). For these reasons, we believe that these oxysulfide composite SEs could represent a completely novel and successful approach to the creation of low-cost, high-energy-density, safe, and long-cycle-life solid-state Na metal batteries.

In summary, a new class of oxysulfide glass SEs with the advantages of both sulfide and oxide SEs was successfully synthesized and systematically examined in both symmetric and full cell configurations. Oxygen-doped sulfide SEs have much stronger and denser glass networks than pure sulfide SEs due to the formation of greater concentrations of oxide and oxysulfide units with BO characteristics. Furthermore, while adding oxygen boosts the mechanical strength of the glassy SE, it also leads these oxysulfide SEs to demonstrate pressure-induced homogeneity of the glassy powder starting materials into homogeneous glass microstructure at ambient temperature. As a result of the greatly increased formability, the oxysulfide SEs exhibit higher mechanical strength and reduced electronic conductivity. Cells exhibit excellent electrochemical stability with Na metal through kinetic stability via the formation of a self-passivating SEI. A homogeneous tri-layer composite SE including Na_3_PS_3.4_O_0.6_|Na_3_PS_3.85_O_0.15_ |Na_3_PS_3.4_O_0.6_ can achieve a critical current density of up to 2.3 mA cm^−2^, the highest recorded CCD value for Na-ion sulfide SEs, and cycle stably at 0.2 mA cm^−2^ for up to 500 h. Na–S full cells were fabricated using a composite oxysulfide bi-layer SE and were demonstrated to have the highest specific energy among solid-state Na–S systems to date. These new oxysulfide SEs, as well as the tri-layer composite SEs that they enable, could pave the way for the development of new glass electrolytes for high-energy, safe, low-cost, and long-cycle-life solid-state batteries in general, and all-solid-state Na–S batteries for energy storage devices in particular.

## Methods

### Synthesis of NPSO SEs

Na_2_S, P_2_S_5_, and P_2_O_5_ (Sigma-Aldrich, 99%) were used as the raw materials without further purification. The Na_3_PS_4−*x*_O_*x*_ (*x* = 0.15, 0.30 and 0.60) SEs were prepared by high-energy planetary ball milling (MTI, MSK-SFM-1). Typically, 2 g mixtures of appropriate amounts of Na_2_S, P_2_S_5_, and P_2_O_5_ powders were milled in an Argon-protected stainless steel jar containing stainless steel milling balls at 500 rpm for 3 h to obtain the amorphous SEs. Note different milling media and milling time were used in this work compared to Lazar et al^16^., which used ZrO_2_-based milling media and 20 h of milling. Ball-milling raw materials in the agate jar at 500 rpm for 20 hours yielded pure sulfide *x* = 0 SE. By heating the glassy Na_3_PS_4_ under vacuum for 2 h at 260 °C, the Na_3_PS_4_ glass-ceramic SE, abbreviated as HT–Na_3_PS_4_, was obtained.

### Materials characterizations of NPSO SEs

Because oxysulfide SEs are sensitive to air and moisture, all of the characterizations were carried out with Argon protection. To determine the crystallinity and the identity of the crystalline phases in the various SEs, lab-based X-ray diffraction (XRD) and synchrotron XRD patterns were collected using a Rigaku MiniFlex 600 with Cu K*α* radiation (*λ* = 1.5418 Å) and Beamline 11–ID–C at Advanced Photon Source with X-ray wavelength of 0.1173 Å, respectively.

The thermal behavior of the SEs powders was examined using differential scanning calorimetry (DSC) on a TA Instruments Q2000. The sample was placed in the Tzero aluminum pan and hermetically crimp-sealed inside the Argon-filled glove box. The DSC measurements were carried out at a heating rate of 20 °C min^−1^ from 50 °C to 400 °C. A Renishaw inVia Raman spectrometer employing a 488 nm Ar^+^ laser and 10 mW of power was used to collect the Raman spectra from 200 to 700 cm^−1^. SE powders were placed into a small plastic sample holder inside the glove box and covered with transparent tape to prevent exposure to air during data collection. Infrared (IR) spectra were acquired on a Bruker IFS 66 v/s vacuum IR spectrometer in the range of 400 − 1200 cm^−1^ using a KBr beamsplitter. During the test, the spectrometer optical bench was held under vacuum to protect the samples from moisture and air. The samples’ IR spectra were obtained by diluting the finely ground glass and glass-ceramic powders to 2% in finely ground and carefully dried CsI and then pressing them into tiny pellets. ^31^P Solid-state Magic Angle Spinning Nuclear Magnetic Resonance (MAS−NMR) spectra were collected using a JEOL ECA-500 NMR spectrometer. SE powders were packed into an alumina spinner with a sealant in an Argon-filled glove box. Spectra were collected using a 4.25 μs, 60° pulse length, and a 200 s recycle delay while rotating at 20 kHz. Chemical shifts were externally referenced to NaH_2_PO_4_.

The elastic modulus *E* and hardness *H* were determined using the same method as previously described^45^. Briefly, *E* and *H* values were measured using a G200 Keysight nanoindenter equipped with a Berkovich tip. All the measurements were conducted inside an argon-filled glove box to eliminate moisture and air contaminations. To assure the convergence of the measured results, indentations with a maximum indentation depth of 1 μm were pressed on different spots of the SE surfaces. During the testing, the load-displacement curves up to pellet cracking were recorded and utilized to calculate the *E* and *H* using the Oliver-Pharr method. $H=\frac{{P}_{{\max }}}{A}$, where *P*_*max*_ is directly determined from the prescribed load (i.e., 1 mN) and the contact area *A* is calibrated as a function of the contact depth. Reduced elastic moduli *E*_*r*_ is derived from the load-displacement response by ${E}_{r}=\frac{S\sqrt{\pi }}{2\sqrt{A}}$, where *S* is the slope of the unloading curve. Elastic modulus *E* is calculated from the *E*_*r*_ by taking into account the deformation of both the indenter and sample $\frac{1}{{E}_{r}}=\frac{1-{v}^{2}}{E}+\frac{\left(1-{v}_{i}^{2}\right)}{{E}_{i}}$ where modulus and Poisson’s ratio of the diamond indenter (*E*_*i*,_ *v*_*i*_) are 1141 GPa and 0.07, respectively; and the Poisson’s ratio *υ* is assumed to be 0.3.

Morphologies of the SEs powders, as well as the surface and cross-section of the densified pellets, were observed using a Gemini LEO 1525 scanning electron microscope (SEM). Nanocrystals embedded in the glass SE were confirmed using a JEOL 2100F transmission electron microscope (TEM). The X-ray photoelectron spectroscopy (XPS) spectra were collected using a Physical Electronics PHI 5700 on the SEs pellets using a monochromatic Mg K*α* X-ray source. The XPS signals were corrected relative to the C 1 *s* signal (284.8 eV) and fitted using the XPSPEAK41 software. The relative density of the SEs was defined as ρ_bulk_/ρ_true_, where ρ_bulk_ is the bulk density and ρ_true_ is the true density of powders according to Nose et al.’s report^18^. ρ_true_ for Na_3_PS_4_ and Na_3_PS_3.4_O_0.6_ were 2.00 g cm^−3^ ^18^ and 2.28 g cm^−3^, respectively.

Time-of-flight secondary mass spectrometry (ToF-SIMS) was performed using a ToF-SIMS NCS instrument, which combines a ToF.SIMS 5 instrument (ION-TOF GmbH, Münster, Germany) and an in-situ Scanning Probe Microscope (NanoScan, Switzerland). A charge compensation with an electron flood gun was applied during the analysis. An adjustment of the charge effects was operated using suitable surface potential and extraction bias for the negative polarity. The cycle time was fixed to 90 µs (corresponding to m/z = 0–737 a.m.u mass range). All samples were transferred under an argon atmosphere with a transfer vessel (IONTOF GmbH) from the glovebox into the analysis chamber. High mass resolution spectra were performed in negative polarity to identify the chemical environment. A bunched 30 keV Bi^3+^ ions (with a measured current of 0.2 pA) was used as primary probe (scanned area 90 × 90 µm^2^) with a raster of 128 × 128 pixels for analysis. The primary ion dose density was limited to 1.00 × 10^12^ ions per cm^2^ for preserving the chemical composition and to achieve comparable measuring conditions between the measurements. A sputter ion beam (Cs^+^ ions at 2 keV energy with a current around 110 nA) with a raster of 300 × 300 µm^2^ was used for high mass resolution depth profile of ToF-SIMS in the negative polarization mode. The analysis area was placed in the center sputter crater to ensure a good quality of the depth profile. The beams were operated in non-interlaced mode, alternating 2 analysis cycles and 20 sputtering cycles followed by a pause of 5 s for charge compensation with an electron flood gun for controlling the sputter/analysis rate ratio. All data evaluation was carried out with the software SurfaceLab 7.0 (IONTOF GmbH).

### Electrochemical characterizations of SEs

The temperature-dependent ionic conductivities of the pelletized SEs were measured from 25 °C to 90 °C using alternating current impedance method (frequency: 1 MHz–0.1 Hz, amplitude: 5 mV) on a VMP3, Bio-Logic Co. electrochemical workstation. Before the measurement, the SEs powders were cold-pressed in a polyetherether-ketone (PEEK) test cell die (φ = 13 mm) under a pressure of 450 MPa and then co-pressed with 20 mg nano-copper powders (Sigma-Aldrich, 40–60 nm, ≥99.5%) as the electrodes under a pressure of 200 MPa. Chemical stability of the SEs towards Na metal was studied by monitoring the impedance change vs. time of the symmetric cell Na|SE|Na, which was assembled by attaching two same pieces of Na metal foils (~100 μm in thickness) on both sides of the SEs. For the fabrication of tri-layer electrolytes, ~150 mg of Na_3_PS_3.85_O_0.15_ glass or Na_3_PS_4_ glass-ceramic SE powders were cold-pressed at 75 MPa; then ~25 mg of Na_3_PS_3.4_O_0.6_ glass powders were evenly distributed on both sides of as-pressed pellet; finally the three layers were co-pressed at 450 MPa. Following the attachment of Na metal foils to the tri-layer electrolyte, a Na plating/stripping test was performed in galvanostatic mode with an initial stacking pressure of 7 MPa.

Since Na_3_PS_3.85_O_0.15_ is the most conductive among the NPSO SEs and Ketjen black carbon (surface area of 1400 m^2^ g^−1^), they were chosen to blend with sulfur to create both fast ionic and electronic pathways for the sulfur active material in the composite cathode. The all-solid-state Na metal-sulfur batteries were fabricated using a Na_3_PS_3.85_O_0.15_|Na_3_PS_3.4_O_0.6_ bi-layer electrolyte, sulfur–Ketjen black–Na_3_PS_3.85_O_0.15_ composite, and Na metal as the separator, cathode, and anode, respectively. In detail, sulfur (99.5%, Alfa Aesar) and Ketjen black (EC-600JD, AkzoNobel) powders with a weight ratio of 1:1 were ball-milled in an agate jar at a rotation speed of 500 rpm for 20 h to obtain a sulfur/KB nanocomposite, which was then milled with Na_3_PS_3.85_O_0.15_ powders at a rotation speed of 350 rpm for 30 min. The weight ratio of sulfur: KB: Na_3_PS_3.85_O_0.15_ composite cathode is 2:2:6. 150 mg Na_3_PS_3.85_O_0.15_ electrolyte powders were firstly pressed at 75 MPa into a pellet, of which one side was then uniformly covered with 20 mg Na_3_PS_3.4_O_0.6_ powders and ~1 mg composite cathode powders. Bulk-type all-solid-state Na–S batteries were fabricated after co-pressing at 450 MPa and attaching a piece of Na metal foil. Galvanostatic tests were performed in the potential range of 1.0 to 3.0 V vs. Na at different current densities from 0.05 to 0.35 mA cm^−2^. All the electrochemical tests were conducted at 60 °C except where otherwise stated.

## Supplementary information

Supplementary Information

## Data Availability

The data that support the plots within this paper and other findings are available from the corresponding authors upon reasonable request.
