# Supplementary material for: An electrochemically stable homogeneous glassy electrolyte formed at room temperature for all-solid-state sodium batteries
Source: Nat Commun. 2022 May 23;13:2854. doi: 10.1038/s41467-022-30517-y (PMC9126868; doi:10.1038/s41467-022-30517-y)
Supplement: Supplementary file 1 — Supplementary Information [file 41467_2022_30517_MOESM1_ESM.pdf]

Supplementary Information

An electrochemically stable homogeneous glassy electrolyte formed at room temperature for all-solid-state sodium batteries

Xiaowei Chi[1,2‡], Ye Zhang[1‡], Fang Hao[1‡], Steven Kmiec[3‡], Hui Dong[1], Rong Xu[4], Kejie Zhao[4], Qing Ai[5], Tanguy Terlier[6], Liang Wang[7†], Lihong Zhao[1], Liqun Guo[1], Jun Lou[5], Huolin L. Xin[8], Steve W. Martin[3*], Yan Yao[1*]

[1]Department of Electrical and Computer Engineering and Texas Center for Superconductivity at the University of Houston, University of Houston, Houston, TX, 77204, USA

[2]Shanghai Institute of Ceramics, Chinese Academy of Sciences, Shanghai 200050, China

[3]Department of Materials Science & Engineering, Iowa State University, Ames, IA 50011, USA

[4]School of Mechanical Engineering, Purdue University, West Lafayette, IN 47907, USA

[5]Department of Materials Science and NanoEngineering, Rice University, Houston, TX 77005, USA

[6]Shared Equipment Authority, SIMS laboratory, Rice University, Houston, TX 77005, USA

[7]Department of Physics, Northern Illinois University, Dekalb, IL 60115, USA

[8]Department of Physics and Astronomy, University of California, Irvine, Irvine, CA 92697, USA

‡ X. C., Y. Z., F. H., S. K. contribute equally to this work.

† Current address: Institute of Advanced Structure Technology and School of Materials Science and Engineering, Beijing Institute of Technology, Beijing 100081, China

[*] Email: yyao4@uh.edu (Y.Y.) swmartin@iastate.edu (S.M.)

Supplementary Table S1 | Assignments of the peaks in the Fourier-transform infrared spectroscopy (FTIR) spectra of $Na_3PS_{4-x}O_x$ SEs

Position (cm^{-1})	Assignment
445	P–S linkage
560	Asymmetric stretching of P–S
590	Stretching of P–O–P
634	Bending vibration of O–P–O
685	Symmetric stretching of P–O–P
720	Symmetric stretching of P–O–P
895	Asymmetric stretching of P–O–P
996	Symmetric stretching of $^{-}$O–P–O^{-} non-bridging oxygen
1030	Motion of the P=O or P–O^{-} non-bridging oxygen
1090	Symmetric stretching of $^{-}$O–P–O^{-} non-bridging oxygen
1150	Symmetric stretching of $^{-}$O–P–O^{-} non-bridging oxygen

Note: P–O connections are also observed in $x = 0$ sample, which may result from the oxygen contamination during synthesis.

Supplementary Table S2 | Fitting results of EIS spectra of $Na_3PS_{4-x}O_x$ (x = 0, 0.15, 0.30, and 0.60) SEs for symmetric cells before and after contact Na metals for 5 hours.

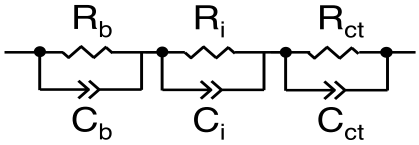

***x* value**	**ASR [Ω·cm²]**	**R_b [Ω·cm²]**	**C_b [pF]**	**R_i [Ω·cm²]**	**C_i [μF]**	**R_{ct} [Ω·cm²]**	**C_{ct} [mF]**
0.00 before	1596.7	1240	1.20	272	0.16	84.7	9.90
0.00 after	1753.0	1350	2.90	304	0.19	99.0	6.30
0.15 before	253.1	216	0.24	6	4.50	31.1	53.0
0.15 after	341.7	254	0.58	16	0.32	71.2	1.53
0.30 before	367.2	334	0.24	24	0.45	8.9	0.45
0.30 after	376.7	332	0.13	28	1.20	16.4	3.30
0.60 before	664.9	644	0.79	-	-	20.9	1.30
0.60 after	662.2	640	0.89	-	-	21.4	1.60

Note: The obtained EIS spectra from high frequency to middle frequency to low frequency can be fitted into bulk resistance and capacitance (R_b + R_{gb}, C_b + C_{gb}) of the SEs, interfacial resistance and capacitance (R_i, C_i) between Na and SEs with characteristic capacitance in the range of 10^{-6}~10^{-7} F, and the charge-transfer resistance and capacitance (R_{ct}, C_{ct}).

Supplementary Table S3 | Comparison of reported solid-state Na–S batteries with this work

Electrolyte	Cathode	Anode	Voltage (V)	Cathode capacity (C_c, mAh g^{-1})	Full cell capacity (C, mAh g^{-1})	Specific energy (Wh kg^{-1})	Cycle life	Ref.
$Na_{3.1}Zr_{1.95}Mg_{0.05}Si_2PO_{12}$ (RT)	S	Na	1.75	527	363	635	10	1
PEO-$NaCF_3SO_3$ (90 °C)	S	Na	1.75	505	352	617	10	2
PEO-$NaN(SO_2F)_2$-1% TiO_2 (60 °C)	S	Na	1.40	713	442	619	100	3
Na_3PS_4 (RT)	S	$Na_{15}Sn_4$	1.10	1522	544	599	2	4
Na_3PS_4 (RT)	S	$Na_{15}Sn_4$	1.30	1112	481	625	25	5
Na_3PS_4 (60 °C)	Na_2S	$Na_{15}Sn_4$	1.25	869	429	536	50	6
Na_3PS_4 (60 °C)	Na_2S	$Na_{15}Sn_4$	1.25	800	411	514	50	7
Na_3SbS_4 (RT)	S	Na_3Sb	0.5	1560	464	232	55	8
**$Na_3PS_{4-x}O_x$ (60 °C)**	**S**	**Na**	**1.42**	**1281**	**610**	**866**	**150**	**This work**
**$Na_3PS_{4-x}O_x$ (25 °C)**	**S**	**Na**	**1.29**	**894**	**506**	**658**	**-**	**This work**

Note: The cell specific energy (E_s) is calculated by $E_s = E \times (C_c^{-1} + C_a^{-1})^{-1}$, where E is the average discharge voltage, C_c is cathode specific capacity. The anode specific capacity (C_a) for Na, $Na_{15}Sn_4$ and Na_3Sb are 1165, 847 and 660 mAh g^{-1}, respectively. The full cell capacity (C) is calculated by $C = (C_c^{-1} + C_a^{-1})^{-1}$.

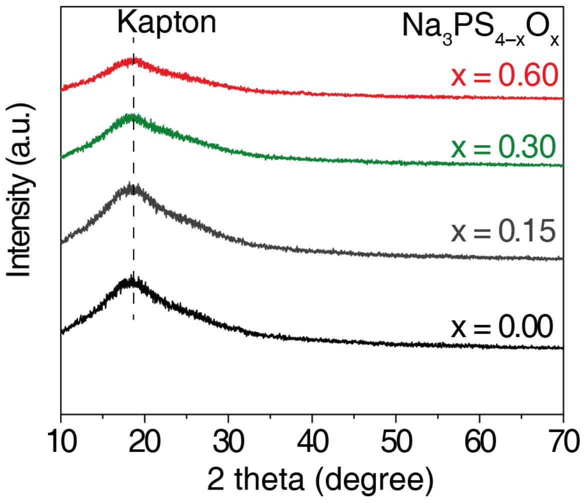

Supplementary Figure 1 | Lab-based X-ray diffraction patterns of amorphous $Na_3PS_{4-x}O_x$ SEs. Note that $x = 0$ sample here is different from HT–Na_3PS_4 that is heat-treated glass-ceramic SE.

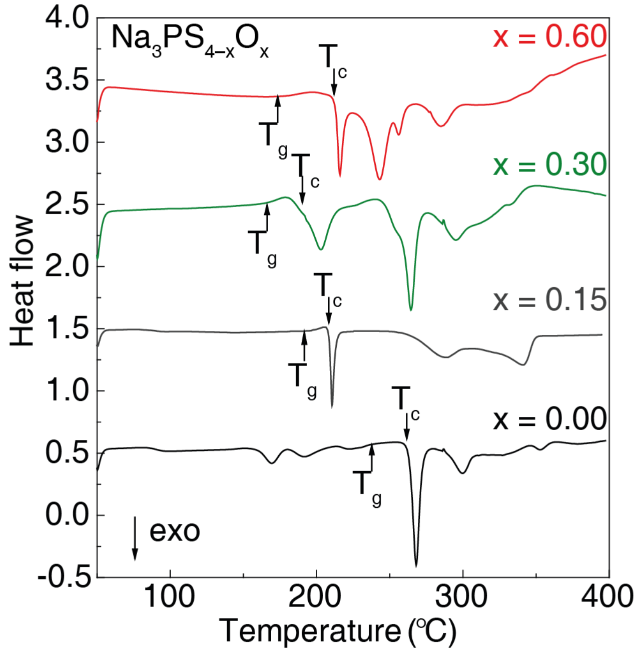

Supplementary Figure 2 | Differential scanning calorimeter (DSC) curves of $Na_3PS_{4-x}O_x$ SEs. T_g and T_c represent the glass transition temperature and crystallization temperature, respectively.

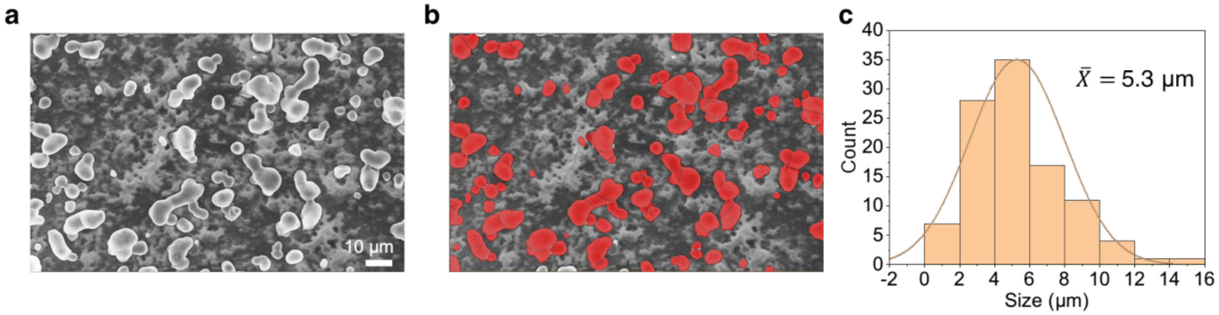

Supplementary Figure 3 | (a-b) SEM images and (c) particle size distribution of $Na_3PS_{3.85}O_{0.15}$ SE powders.

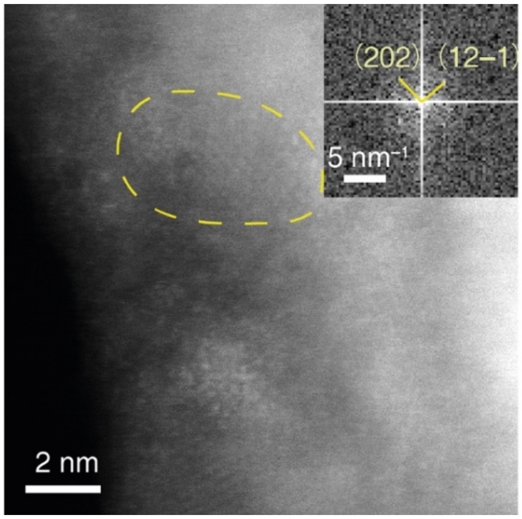

Supplementary Figure 4 | Transmission electron microscope (TEM) image and fast Fourier transform (FFT) patterns of $Na_3PS_{3.85}O_{0.15}$ SE.

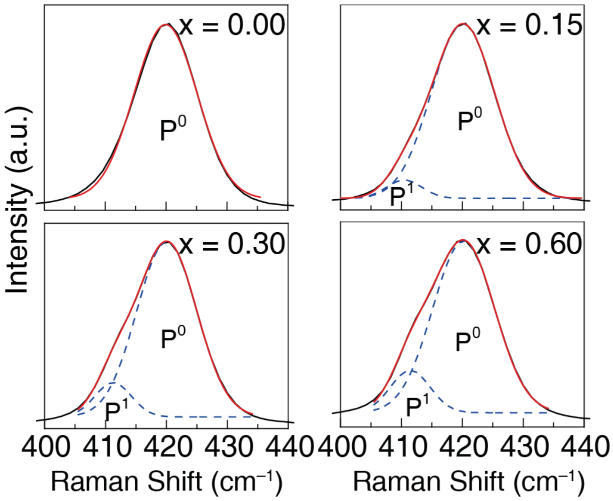

Supplementary Figure 5 | Gaussian fitting results of PS_4 mode in Raman spectra of $Na_3PS_{4-x}O_x$ SEs.

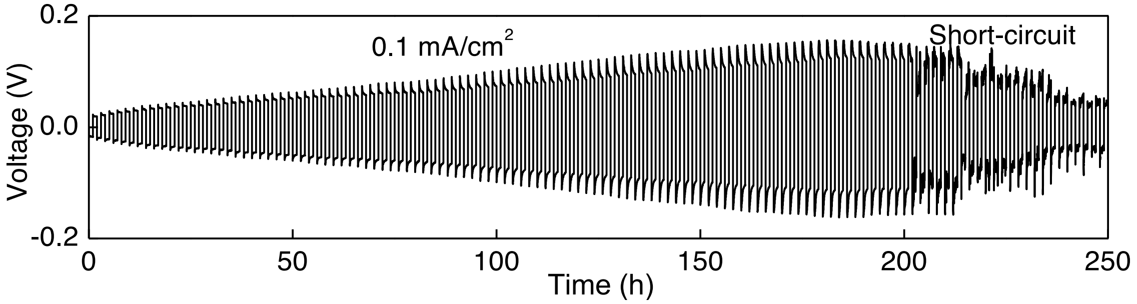

Supplementary Figure 6 | Galvanostatic cycling of Na|HT–Na_3PS_4|Na symmetric cells at 60 °C under current density of 0.1 mA cm^{-2} with a stripping/plating capacity of 0.1 mAh cm^{-2}.

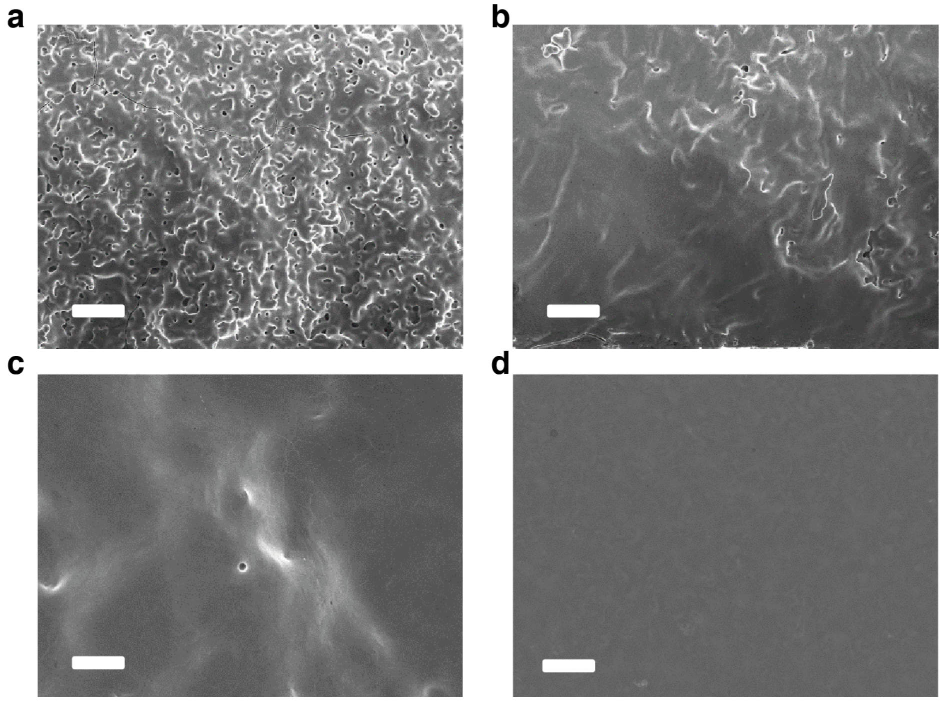

Supplementary Figure 7 | SEM images of cross-sectional Na_3PS_4 glass SE pressed at (a) 150 MPa and (b) 375 MPa, in comparison with $Na_3PS_{3.4}O_{0.6}$ glass SE pressed at (c) 150 MPa and (d) 375 MPa (scale bar: 20 μm).

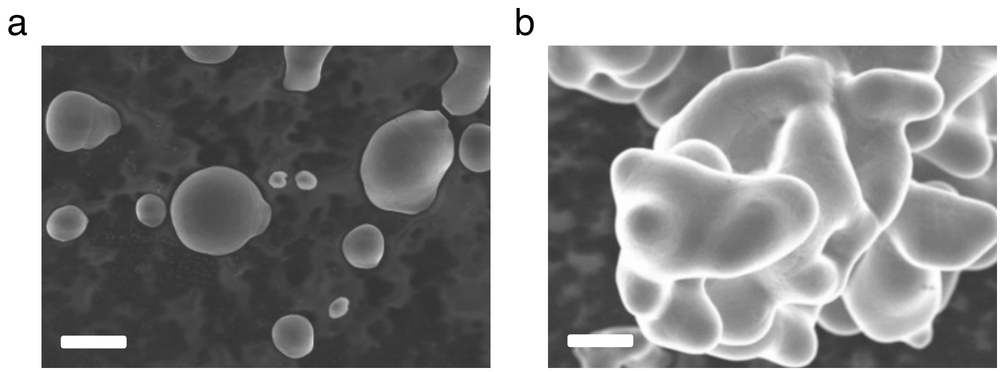

Supplementary Figure 8 | SEM images of (a) Na_3PS_4 glass and (b) $Na_3PS_{3.4}O_{0.6}$ glass powders (scale bar: 5 μm).

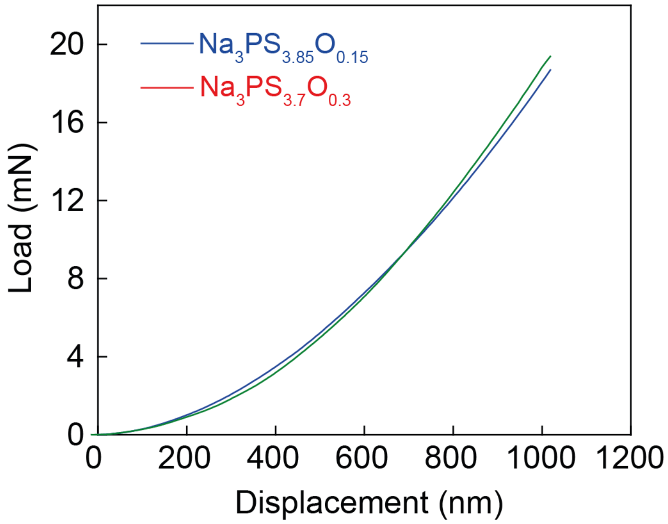

Supplementary Figure 9 | Nanoindentation load-displacement curves of $Na_3PS_{3.85}O_{0.15}$ and $Na_3PS_{3.7}O_{0.3}$ glass SEs.

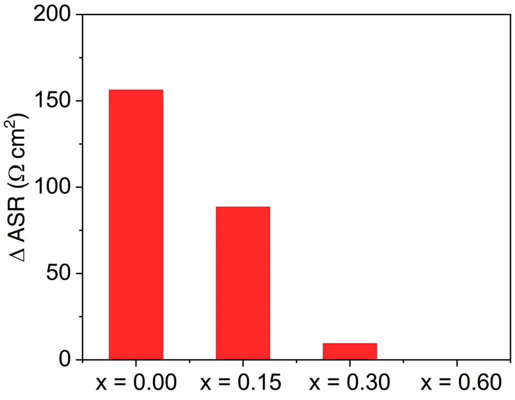

Supplementary Figure 10 | Areal Specific Resistance (ASR) change of $Na_3PS_{4-x}O_x$ SEs before and after contacting with Na metal for 5 hours.

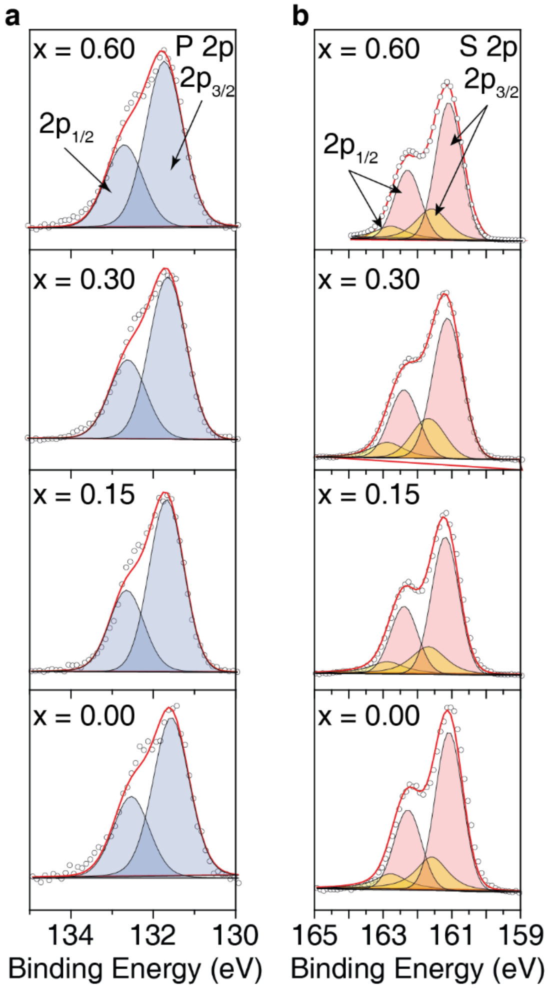

Supplementary Figure 11 | P 2*p* and S 2*p* orbitals in XPS spectra of pristine $Na_3PS_{4-x}O_x$ SEs.

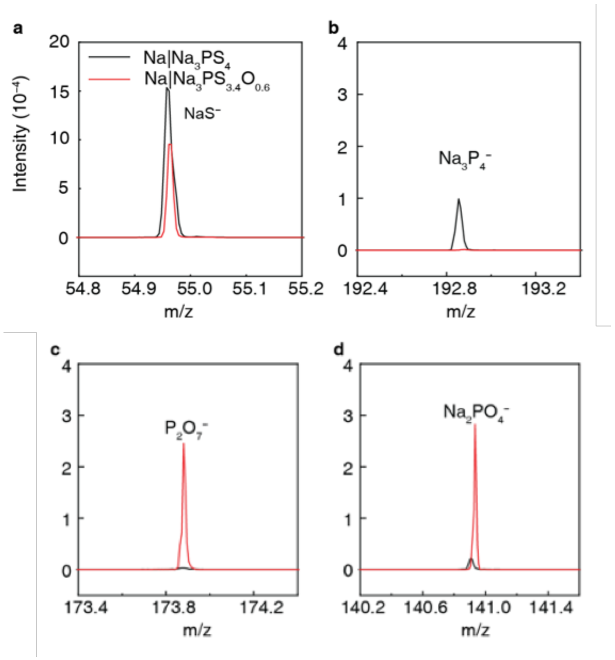

Supplementary Figure 12 | ToF-SIMS spectra for Na|Na_3PS_4 and Na|$Na_3PS_{3.4}O_{0.6}$ interface using Bi^{3+} primary ions and Cs^+ sputtering ions.

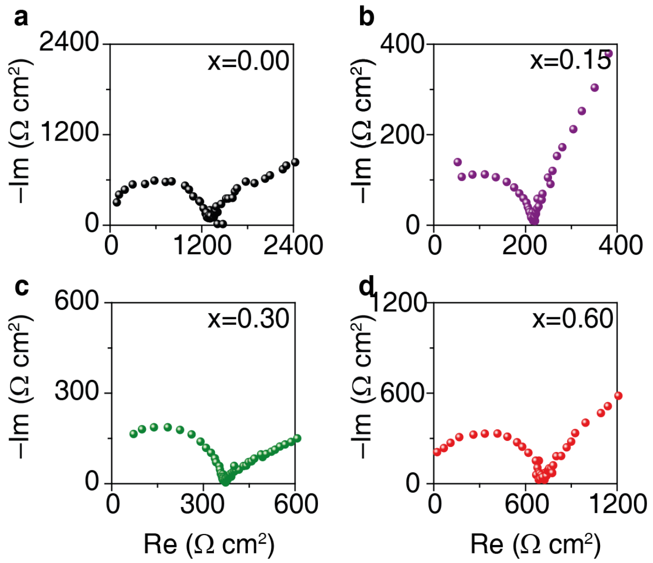

Supplementary Figure 13 | Electrochemical impedance spectra (EIS) of $Na_3PS_{4-x}O_x$ SEs in Cu|SE|Cu cells at 60 °C.

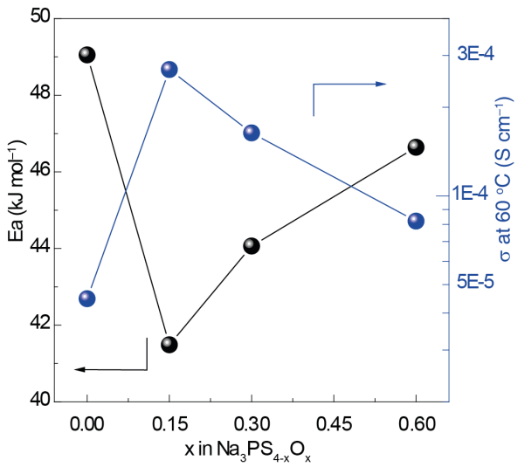

Supplementary Figure 14 | Plots of activation energies (E_a) and conductivity of $Na_3PS_{4-x}O_x$ SEs at 60 °C.

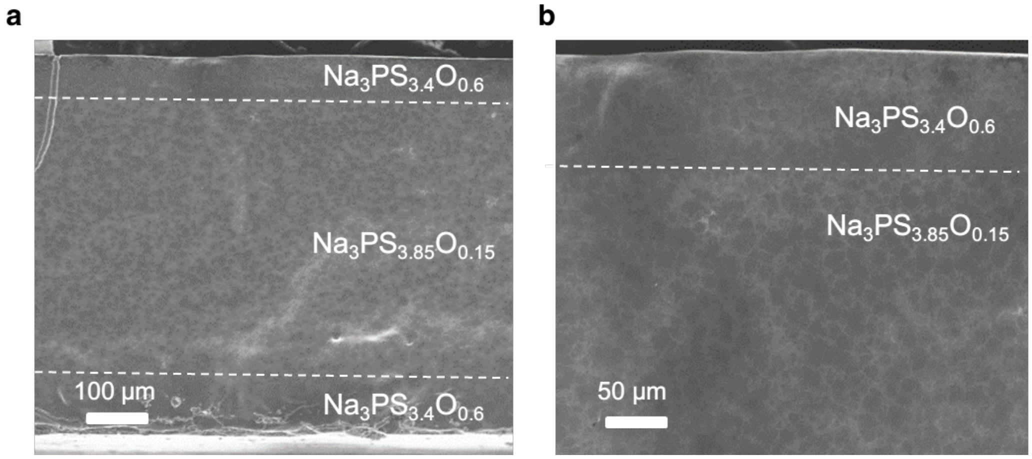

Supplementary Figure 15 | Cross-sectional SEM images of the tri-layer electrolyte.

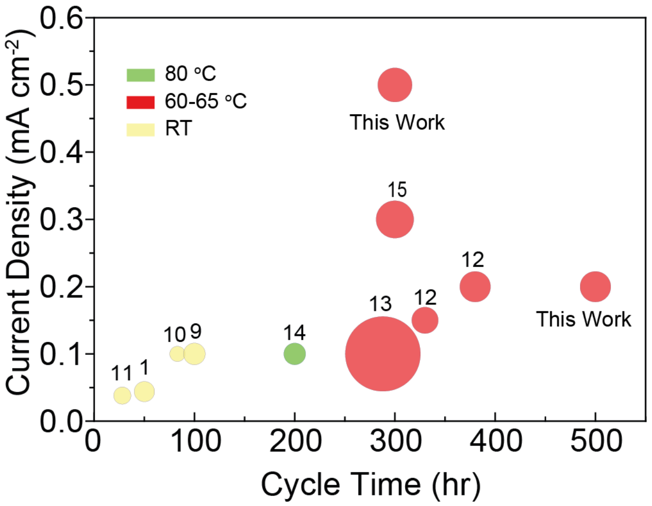

Supplementary Figure 16 | Comparison of reported Na|SE|Na symmetric cell performance with this work in terms of the capacity of Na metal plated per cycle (circle area), current density, cycling time. SEs include sulfide-based (Na_3PS_4, Na_3SbS_4, $Na_{2.9375}PS_{3.9375}Cl_{0.0625}$)[9-11], oxide-based ($Na_{3.1}Zr_{1.95}Mg_{0.05}Si_2PO_{12}$, heat-treated $Na_3Zr_2(PO_4)(SiO_4)_2$)[1,12], boride-based ($Na_2(B_{12}H_{12})_{0.5}(B_{10}H_{10})_{0.5}$)[13], polymer-based (poly(ethylene oxide) (PEO), hollow mesoporous organic polymer-PEO composite)[14,15], and polymer-oxide composite (cross-linked poly(ethylene glycol) methyl ether acrylate (CPMEA)–$Na_3Zr_2(PO_4)(SiO_4)_2$)[12].

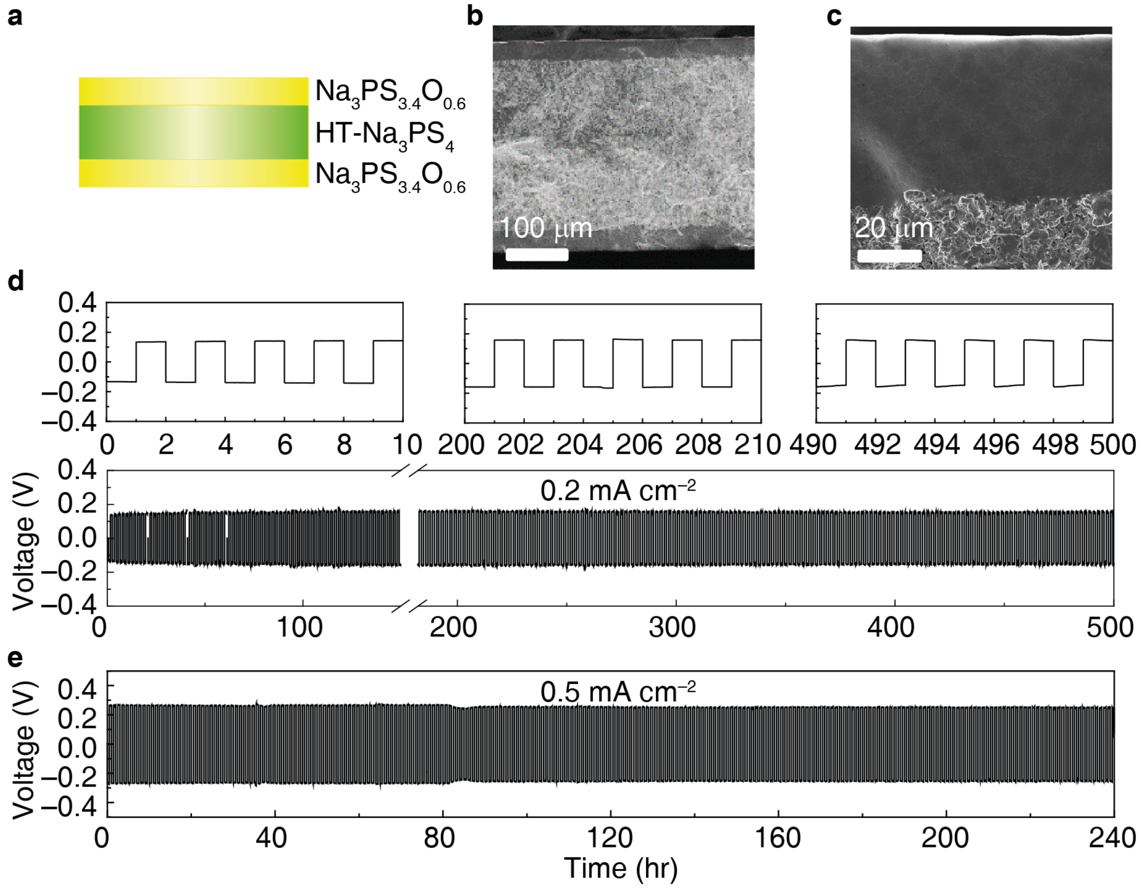

Supplementary Figure S17 | Symmetric cell performances: (a) Scheme of tri-layer electrolyte composed of $Na_3PS_{3.4}O_{0.6}$|HT–Na_3PS_4|$Na_3PS_{3.4}O_{0.6}$; (b) SEM image of the tri-layer electrolyte; (c) SEM image of enlarged interface between $Na_3PS_{3.4}O_{0.6}$ (top layer) and HT–Na_3PS_4 (bottom layer); (d) and (e) galvanostatic cycling of Na|$Na_3PS_{3.4}O_{0.6}$|HT–Na_3PS_4|$Na_3PS_{3.4}O_{0.6}$|Na symmetric cells at 60 °C under current densities of 0.2 mA cm^{-2} and 0.5 mA cm^{-2}, respectively.

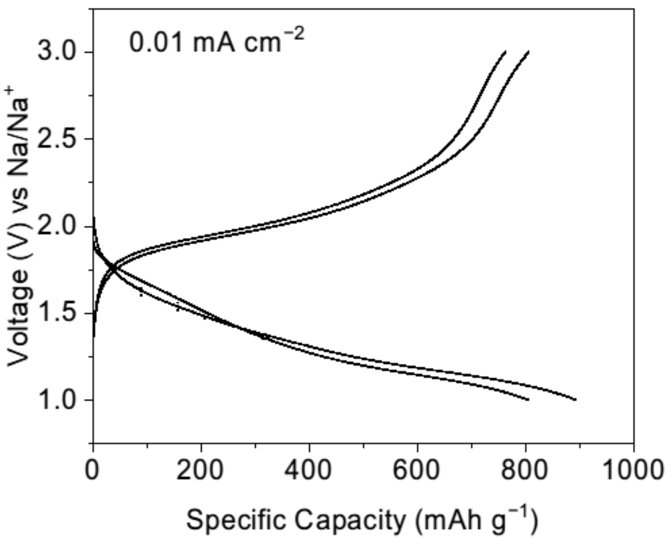

Supplementary Figure 18 | All-solid-state Na–S full cell based on the oxysulfide SEs measured at room temperature.

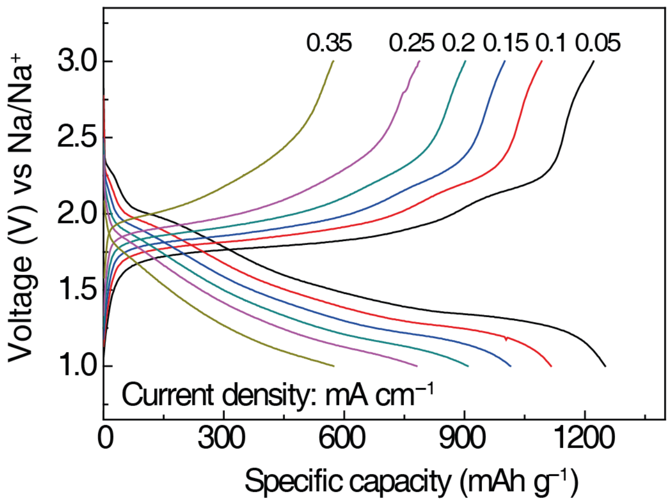

Supplementary Figure 19 | Voltage profiles of Na–S full cells at 60 °C under current densities from 0.05 to 0.35 mA cm^{-2}.

References

1. Song, S., Duong, H. M., Korsunsky, A. M., Hu, N. & Lu, L. A Na+ Superionic Conductor for Room-Temperature Sodium Batteries. *Sci. Rep.* **6**, 32330, (2016).
2. Park, C.-W. *et al.* Discharge properties of all-solid sodium-sulfur battery using poly(ethylene oxide) electrolyte. *J. Power Sources* **165**, 450-454, (2007).
3. Zhu, T. *et al.* An All-Solid-State Sodium–Sulfur Battery Using a Sulfur/Carbonized Polyacrylonitrile Composite Cathode. *ACS Applied Energy Materials* **2**, 5263-5271, (2019).
4. Nagata, H. & Chikusa, Y. An All-solid-state Sodium-Sulfur Battery Operating at Room Temperature Using a High-sulfur-content Positive Composite Electrode. *Chem. Lett.* **43**, 1333-1334, (2014).
5. Tanibata, N., Deguchi, M., Hayashi, A. & Tatsumisago, M. All-Solid-State Na/S Batteries with a Na3PS4 Electrolyte Operating at Room Temperature. *Chem. Mater.* **29**, 5232-5238, (2017).
6. Yue, J. *et al.* High-Performance All-Inorganic Solid-State Sodium–Sulfur Battery. *ACS Nano* **11**, 4885-4891, (2017).
7. Fan, X. *et al.* High-Performance All-Solid-State Na–S Battery Enabled by Casting–Annealing Technology. *ACS Nano* **12**, 3360-3368, (2018).
8. Ando, T., Sakuda, A., Tatsumisago, M. & Hayashi, A. All-solid-state sodium-sulfur battery showing full capacity with activated carbon MSP20-sulfur-Na3SbS4 composite. *Electrochem. Commun.* **116**, 106741, (2020).
9. Wan, H. *et al.* Nanoscaled Na3PS4 Solid Electrolyte for All-Solid-State FeS2/Na Batteries with Ultrahigh Initial Coulombic Efficiency of 95% and Excellent Cyclic Performances. *ACS Appl. Mater. Interfaces* **10**, 12300-12304, (2018).
10. Wang, H. *et al.* An Air-Stable Na3SbS4 Superionic Conductor Prepared by a Rapid and Economic Synthetic Procedure. *Angew. Chem. Int. Ed.* **55**, 8551-8555, (2016).
11. Wu, E. A. *et al.* New Insights into the Interphase between the Na Metal Anode and Sulfide Solid-State Electrolytes: A Joint Experimental and Computational Study. *ACS Appl. Mater. Interfaces* **10**, 10076-10086, (2018).
12. Zhou, W., Li, Y., Xin, S. & Goodenough, J. B. Rechargeable Sodium All-Solid-State Battery. *ACS Cent. Sci.* **3**, 52-57, (2017).
13. Duchêne, L. *et al.* A highly stable sodium solid-state electrolyte based on a dodeca/deca-borate equimolar mixture. *Chem. Commun.* **53**, 4195-4198, (2017).
14. Ma, Q. *et al.* A new Na[(FSO2)(n-C4F9SO2)N]-based polymer electrolyte for solid-state sodium batteries. *J. Mater. Chem. A* **5**, 7738-7743, (2017).
15. Zhou, W., Gao, H. & Goodenough, J. B. Low-Cost Hollow Mesoporous Polymer Spheres and All-Solid-State Lithium, Sodium Batteries. *Adv. Energy Mater.* **6**, 1501802, (2016).
